# Comparing oblique lumbar interbody fusion with lateral screw fixation and percutaneous endoscopic transforaminal discectomy (OLIF-PETD) and minimally invasive transforaminal lumbar interbody fusion (MIS-TLIF) for the treatment of lumbar disc herniation complicated with lumbar instability

**DOI:** 10.1186/s12891-022-06075-1

**Published:** 2022-12-19

**Authors:** Chen Deng, Haoyu Feng, Xun Ma, Chen Chen, Jun Mei, Lin Sun

**Affiliations:** 1 Department of Orthopedics, Third Hospital of Shanxi Medical University, Shanxi Bethune Hospital, Shanxi Academy of Medical Sciences, Tongji Shanxi Hospital, 030032 Taiyuan, China; 2 Department of Orthopedics, Tongji Hospital, Tongji Medical College, Huazhong University of Science and Technology, Wuhan, 430030 China

**Keywords:** Oblique lumbar interbody fusion, Percutaneous endoscopic transforaminal discectomy, Lateral screw fixation, Minimally invasive spine surgery, Multifidus

## Abstract

**Objective:**

To evaluate the early clinical effect of oblique lumbar interbody fusion with lateral screw fixation and percutaneous endoscopic transforaminal discectomy (OLIF-PETD) in the treatment of lumbar disc herniation with lumbar instability.

**Methods:**

A total of 22 patients with lumbar disc herniation and lumbar instability from August 2017 to August 2019 were enrolled in this retrospective study. The general information, perioperative indicators and complications were recorded. The clinical outcomes and radiological outcomes were evaluated before the operation, seven days after the operation, and at the last follow-up. Vertebral fusion and degree of multifidus muscle injury were evaluated at the last follow-up.

**Results:**

In this study, OLIF + PETD showed shorter incision length compared to the MIS-TLIF (*P* < 0.001). In the two groups, the clinical outcomes and radiological outcomes were significantly improved compared with the preoperative (*P* < 0.001). At the seven days after the operation and the last follow-up, the VAS of OLIF + PETD group was lower than that of MIS-TLIF group (*P* < 0.05). OLIF + PETD could give superior outcome in restoring disc height (*P* < 0.001), but the fusion segment angle of OLIF + PETD group was larger compared to the MIS-TLIF group seven days after the operation and at the last follow-up (*P* < 0.05). In addition, the fusion rate was not significantly different between the two groups (*P* > 0.05), but OLIF + PETD could avoid the multifidus injury (*P* < 0.001).

**Conclusion:**

Compared to MIS-TLIF, OLIF-PETD can achieve satisfactory decompression effects and fusion rates with less multifidus injury and postoperative low back pain, which may be an alternative choice for the treatment of lumbar disc herniation combined with lumbar instability.

## Introduction

Lumbar disc herniation (LDH) is an important cause of low back pain and lower limb pain in the clinic. This kind of patient is often complicated with lumbar instability, which leads to low back pain and unilateral lower limb symptoms. Traditional LDH with lumbar instability is often treated with posterior lumbar interbody fusion (PLIF) and transforaminal lumbar interbody fusion (TLIF). These two operations cause great damage to the normal stability structure of the lumbar spine and may cause intractable low back pain after the operation and affect the effect of fusion [1]. In recent years, minimally invasive transforaminal lumbar interbody fusion (MIS-TLIF) has achieved good clinical results in the treatment of LDH, lumbar instability and other lumbar degenerative diseases [2]. However, recent studies have reported that the early clinical efficacy of MIS-TLIF is still controversial [3, 4].

Oblique lumbar interbody fusion (OLIF) was introduced by Silvestre [5] in 2012 and has been applied to lumbar spinal stenosis, lumbar instability and other degenerative diseases in recent years with satisfactory clinical results. However, OLIF achieves indirect decompression by expanding the height of the intervertebral space and the area of the intervertebral foramen. Its limited decompression and inapplicability to the L_5_-S_1_ limit its application. A large number of recent articles have confirmed the effectiveness of percutaneous endoscopic transforaminal discectomy (PETD) in the treatment of LDH and concluded that the incidence of perioperative complications of PETD is low [6]. Therefore, we used OLIF with lateral screw fixation (LSF) and PETD (OLIF-PETD) for the treatment of LDH combined with lumbar instability, with satisfactory early clinical efficacy.

This retrospective study aimed to evaluate the early clinical effect and decompression effect of OLIF-PETD in the treatment of LDH with lumbar instability and provide more reference for the selection of surgery for such patients.

## Material and methods

### Study design and settings

The present study is a Scientific Research Ethics Committee-approved retrospective analysis of patients treated with OLIF-PETD and MIS-TLIF for LDH complicated with lumbar instability at our institution (single-surgical team) between 2017 and 2019. All patients had symptoms of low back pain, accompanied by lower limb pain or numbness. All patients underwent X-ray, CT three-dimensional reconstruction and MRI examinations before surgery.

### Participants and eligibility criteria

All patients with a diagnosis of LDH with lumbar instability who underwent spinal surgery at our hospital between 2017 and 2019 were potentially to be eligible for this study.

Inclusion Criteria: ① The patient was diagnosed with LDH complicated with lumbar instability (L_2_-L_5_): lumbar hyperextension and flexion lateral radiographs with sagittal displacement > 4 mm or intervertebral angle greater than 10° [7]; the symptoms involve lumbago and back pain combined with unilateral lower limb nerve compression symptoms, and strict conservative treatment is ineffective, requiring surgical intervention; ② OLIF with LSF and PETD or MIS-TLIF was used for treatment; ③ the main outcome measures were clinical efficacy and radiological measurement.

Exclusion Criteria: ①Complicated with spondylolisthesis or lumbar spondylolisthesis (Meyerding Grade ≥ 2); ②Complicated with a history of lumbar surgery; ③Complicated with spinal tumor, tuberculosis, infection, vertebral fracture or deformity; ④Complicated with severe osteoporosis.

### Surgical method

**Fig. 1:**
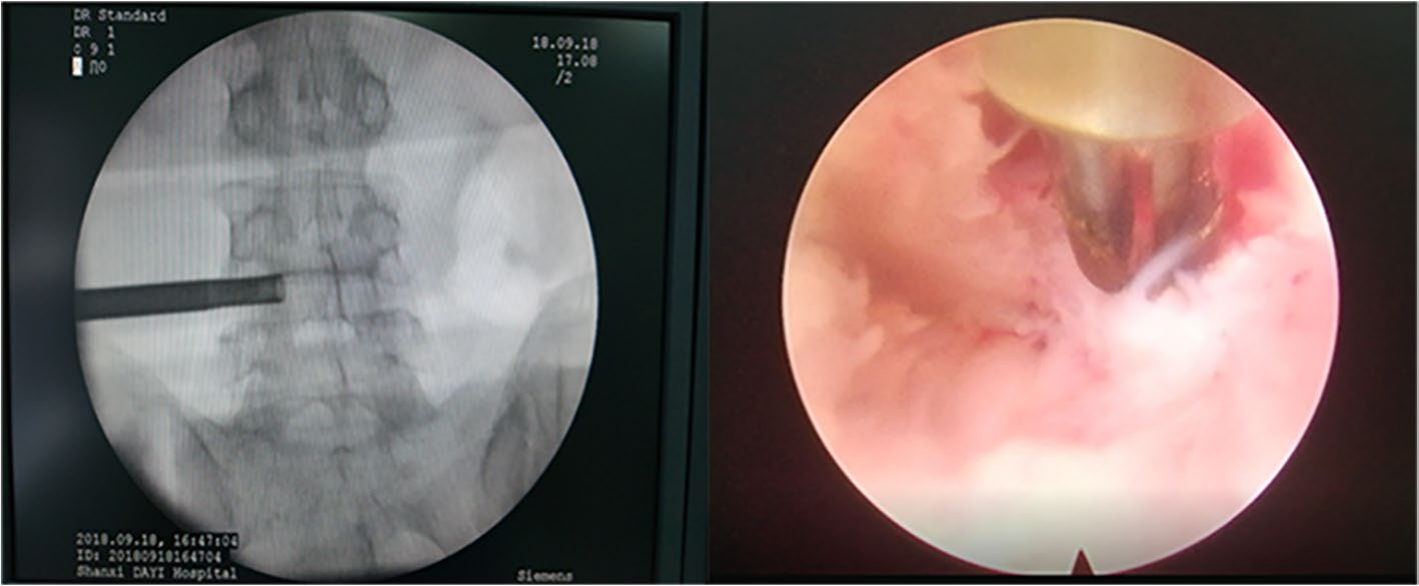
PETD Direct decompression under percutaneous endoscopy

OLIF + PETD: the PETD procedure was performed first, with the patient lying prone on the operating table under local anesthesia. Under G-arm fluoroscopy, the guide needle was punctured into the vertebral superior articular process, and the angle of 15–30° was the safe area. The skin cut was made (8 mm), the incision was dilated by a dilator and working channel. Intervertebral foramen plasty was performed with a ring saw, and a spinal endoscope was inserted (SPINENDOS GmbH, Munich, Germany). Different types of nucleus pulposus forceps are used to remove the herniated nucleus pulposus. At the same time, bipolar radiofrequency ablation (SPINENDOS GmbH, Munich, Germany) was used for hemostasis and to form the annulus fibrosus. After decompression, the endoscopic working sleeve was removed (Fig. 1).

**Fig. 2:**
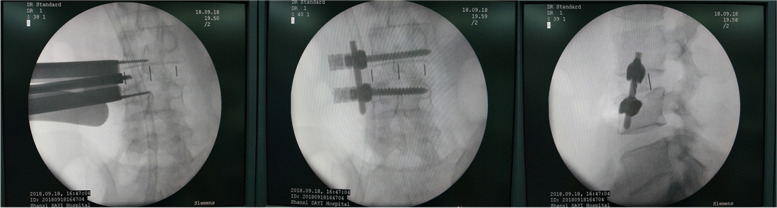
OLIF OLIF combined with LSF

The patient was then placed in the right decubitus position under general anesthesia for OLIF. Under G-arm fluoroscopy, the intervertebral space and anterior edge of the responsible vertebral body were located. Expose the intervertebral space and lateral side of the vertebral body. The insertion channel will be properly extended to open the intervertebral space. The anterior 1/3 annulus fibrosus was incised, and nucleus pulposus forceps and reamers were used alternately to remove intervertebral disc tissue. After discectomy and exposure of the bony endplate, a suitably sized cage was inserted and filled with allogeneic bone and bone repair materials. The multiaxial universal screw was screwed in the lateral side of the vertebral body with the screw passing through the contralateral cortex as far as possible. The longitudinal connecting rod and tail cap were placed (Fig. 2). Finally, the incision was closed layer by layer.

MIS-TLIF: under general anesthesia, the patient was placed in the prone position. Under G-arm fluoroscopy, the pedicle of the responsible vertebral body was marked on the body surface. A puncture needle was used to locate the vertebral space on the line of the pedicle marker point on the side of the disc herniation. A longitudinal incision was made (4 cm) and the working channel was inserted step by step. The nerve roots and thecal sac were exposed with osteotome and rongeur. A thorough discectomy was performed to expose the bony endplate. Then a cage filled with autogenous bone and bone repair materials was inserted. The pedicle screw guide needle was placed bilaterally, the hollow universal screw was screwed, and the prebent connecting rod was placed. A drainage tube was placed in the wound, and the incision was closed.

### Outcome measures

1. Demographic data: Age, sex, and intraoperative parameters, including fusion level, operative duration, incision length, hospital stays, intraoperative blood loss, postoperative drainage, and complications.
2. Clinical efficacy evaluation:

Visual analog scale (VAS): Assess the overall pain in the waist, lower extremities, and surgery. VAS scores were independently filled in by patients after the doctor's brief explanation, with 0 being no pain and 10 being very pain.

Japanese Orthopedic Association assessment of treatment score (JOA): Lumbar function was assessed at preoperative and postoperative follow-up. The total lumbar JOA score was 29, including symptoms, physical signs and bladder function. JOA improvement rate = (follow-up score — preoperative score)/(29 — preoperative score) × 100%.

Oswestry disability index (ODI): The subjective function of the lumbar spine was assessed with a total score of 100, including pain degree, daily living, self-care ability, lifting, walking, sitting, standing, sleeping, sexual life, social activities, travel, etc. ODI = each score/total score × 100%.

3. Degree of lumbar multifidus muscle injury

**Fig. 3:**
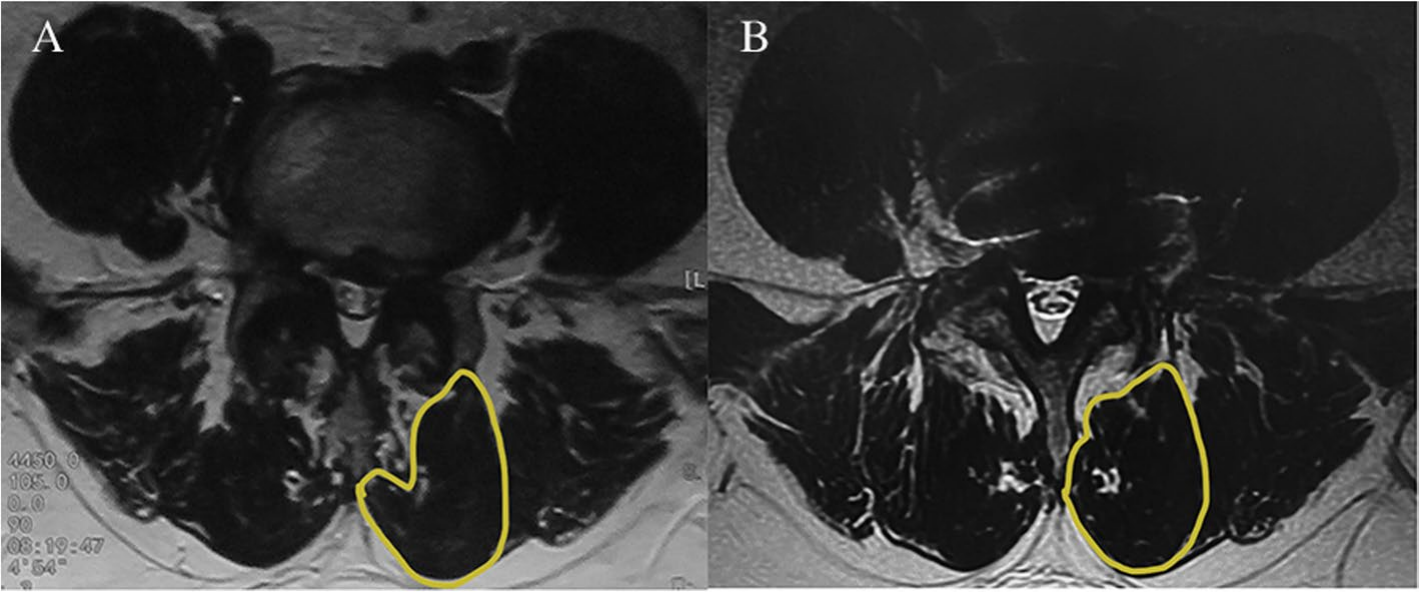
Measurement of lumbar multifidus muscle cross-sectional area (MF-CSA). **A** Preoperative MF-CSA. **B** The last follow-up MF-CSA

The multifidus muscle cross-sectional area (MF-CSA) of the affected side was measured before the operation and at the last follow-up on the MRI axial image of the lumbar vertebrae. The innermost fascia close to the outer edge of the spinous process and lamina was taken as the lateral boundary of the multifidus muscle, fat infiltration was excluded, and ImageJ software was used for measurement (Fig. 3). Atrophy rate = (preoperative CSA — last follow-up CSA)/preoperative CSA × 100%.

4. Radiological measurement [8]

**Fig. 4:**
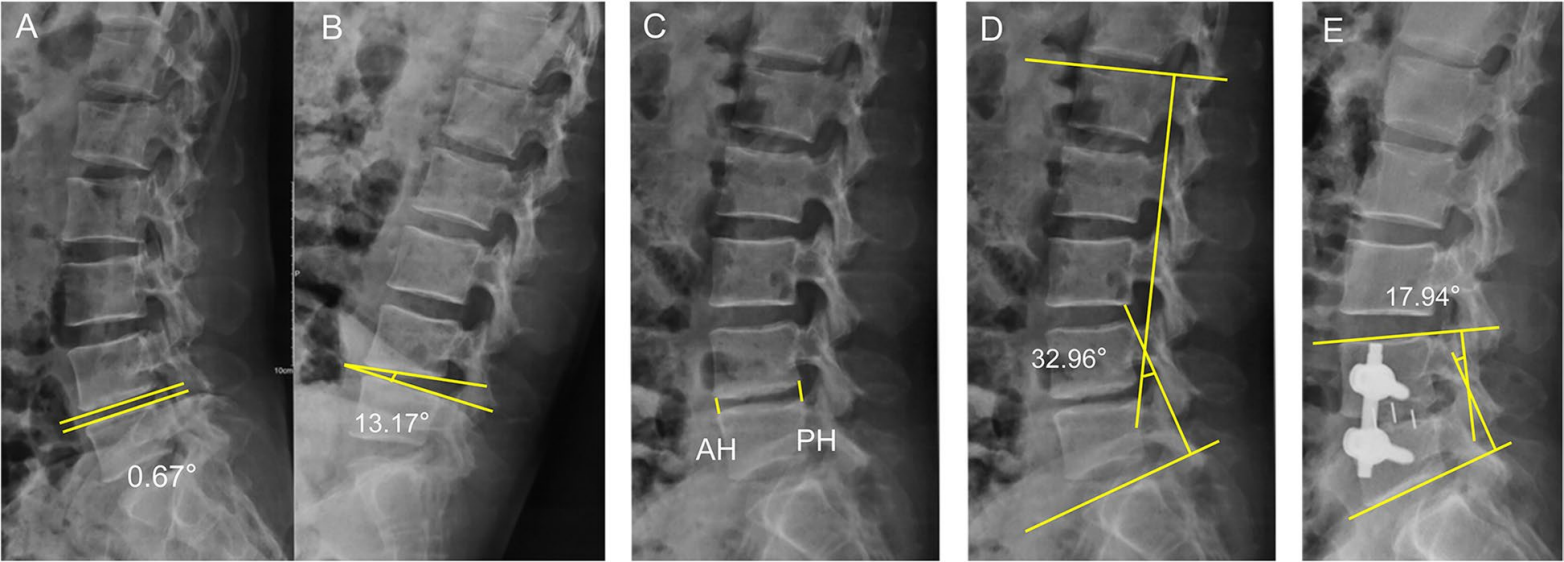
X-ray related index measurement. **A**, **B** Preoperative lumbar hyperextension and flexion lateral radiographs showed a change in intervertebral space angulation greater than 10°, which was consistent with the diagnosis of lumbar instability. **C** Lumbar lateral radiography DH measurement DH = (AH + PH)/2. **D** Lumbar lateral radiography LA measurement. **E** FSA measurement on lateral lumbar radiography

Lumbar spine lateral X-ray measurement indexes were as follows. Disc height (DH): The anterior vertebral height (AH) and posterior vertebral height (PH) of the fusion segment were measured in lateral X-ray, DH (mm) = (AH + PH)/2 mm; lumbar lordosis angle (LA): LA was measured on lateral X-ray with two measurement lines parallel to the L1 and S1 upper endplates; fusion stage angle (FSA): the angle between the upper endplate of the upper vertebral body and the lower endplate of the lower vertebral body of the fusion segment was measured by lateral X-ray (Fig. 4).

**Fig. 5:**
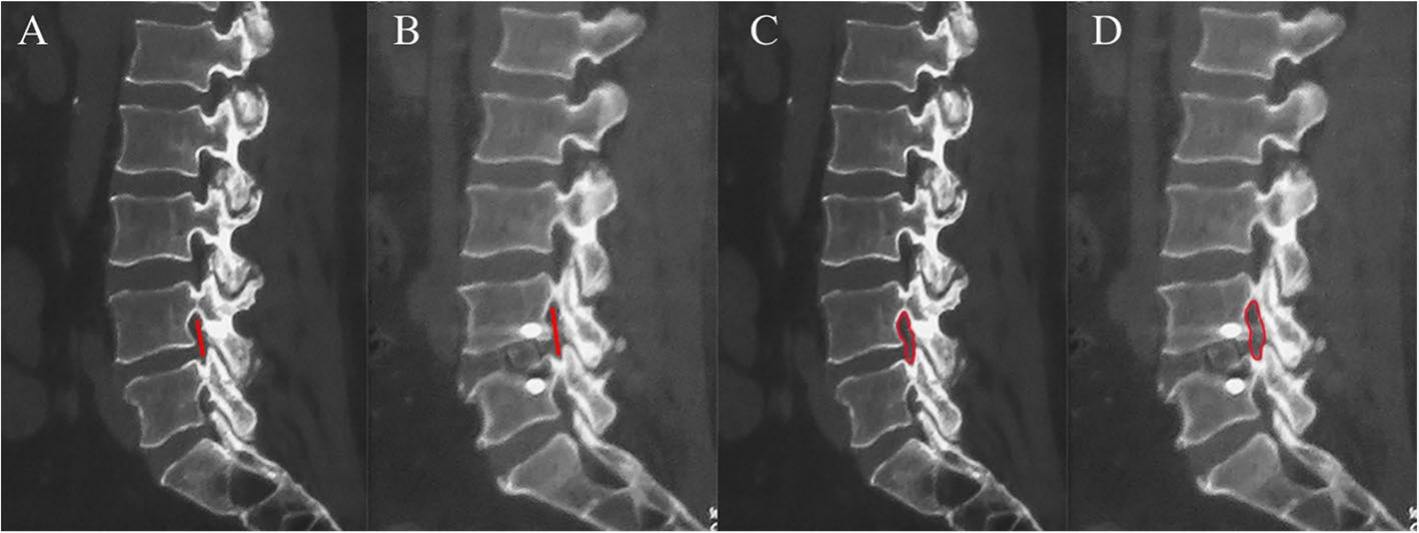
CT-related index measurement. **A**, **B** The L_4-5_ FH measured on preoperative (**A**) and postoperative (**B**) sagittal CT scan images: the shortest distance between the lower edge of the upper pedicle and the upper edge of the upper pedicle. **C**, **D** The L_4-5_ F-CSA was measured on preoperative (**C**) and postoperative (**D**) sagittal CT scan images

Lumbar spine computed tomography (CT) measurement indexes were as follows. Foraminal height (FH): the shortest distance between the lower edge of the upper vertebral pedicle and the upper edge of the lower vertebral pedicle, measured on lumbar CT sagittal image; foraminal cross-sectional area (F-CSA): measured on lumbar CT sagittal image, the area of the nerve root outlet region after excluding soft tissues such as discs, joint capsule and surrounding bone structures for the foraminal area (Fig. 5).

**Fig. 6:**
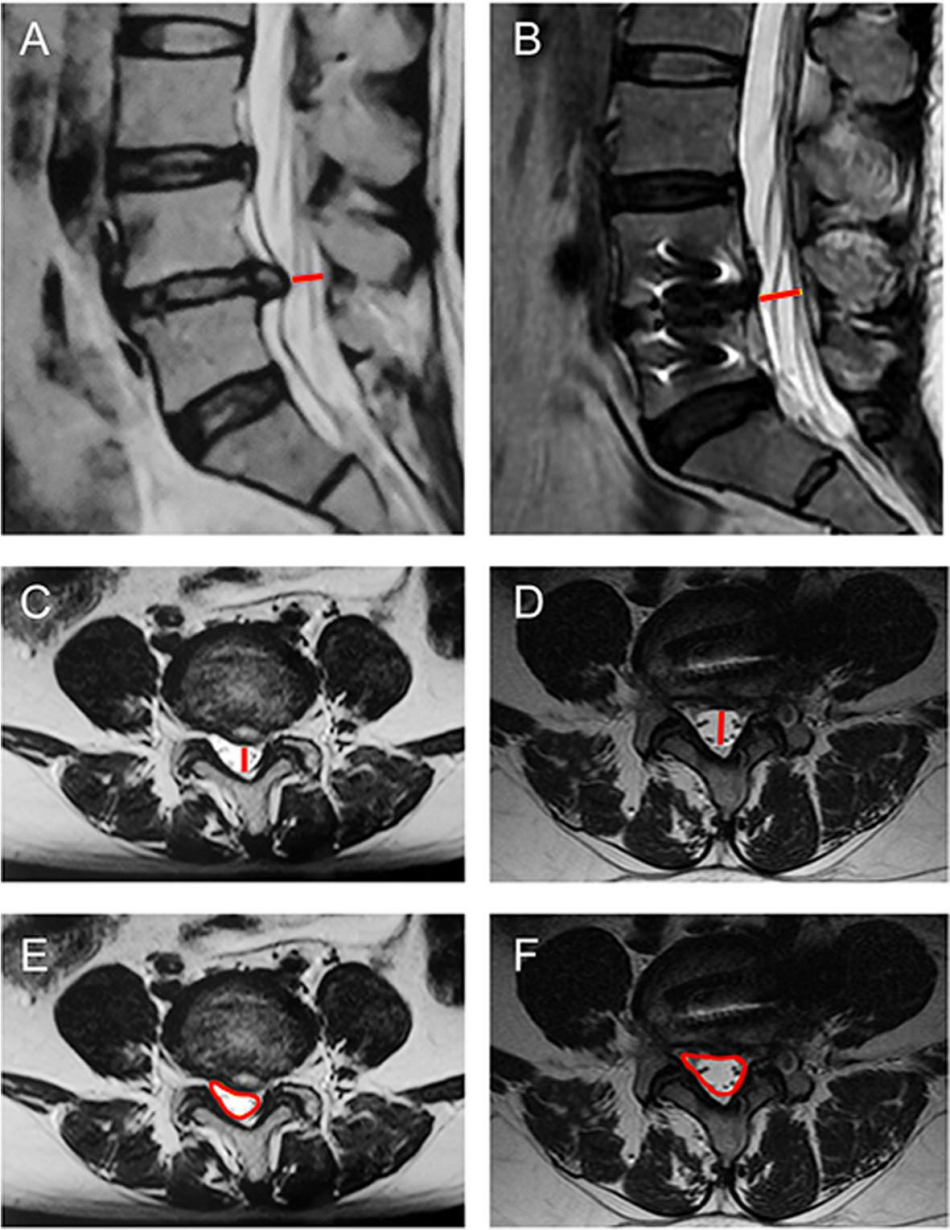
Measurement of MRI-related indexes. **A**, **B** SSCD was measured on preoperative (**A**) and postoperative (**B**) lumbar MRI sagittal images: anterior and posterior length of the spinal canal at the central level of the intervertebral space. **C**, **D** ASCD was measured on preoperative (**C**) and postoperative (**D**) lumbar MRI axial images: the length of the spinal canal at the anterior and posterior levels. **E**, **F** SC-CSA was measured on preoperative (**E**) and postoperative (**F**) lumbar MRI axial images

Lumbar spine magnetic resonance imaging (MRI) measurement indexes were as follows. Sagittal spinal canal diameter (SSCD): measured on MRI sagittal image of the lumbar spine, it is the anterior and posterior length of the spinal canal at the central level of the intervertebral space excluding the anterior intervertebral disc and posterior fat tissue, ligamentum flavum and other soft tissues; axial spinal canal diameter (ASCD): the length of the spinal canal in the horizontal direction measured on axial MRI images of the lumbar spine after excluding bony structures and soft tissues such as intervertebral discs, ligamentum flavum and fat tissue in the spinal canal; spinal canal cross-sectional area (SC-CSA): measured on axial MRI images of the lumbar spine, also excluding the bony structures and soft tissue such as intervertebral discs, ligamentum flavum and fat tissue in the spinal canal (Fig. 6).

Radiological measurement data were measured using ImageJ software. Radiological measurement data were independently performed by three authors (JM, LS and CD.).

5. Fusion assessment at the last follow-up

A CT scan was performed at the last follow-up, and interbody fusion was divided into three levels according to BSF grading [9]. BSF-1: vertebral collapse, loss of vertebral space height, vertebral slippage, loosening of internal fixation or obvious absorption and subsidence of bone graft, visible light transmission around the fusion apparatus; BSF-2: continuous bone trabeculae connecting upper and lower endplates were seen in the fusion apparatus, and transparent bands were completely transverse; BSF-3: there were fully continuous bone trabeculae connected to the upper and lower endplates of the vertebral body inside or outside the fusion apparatus, and extensive osteogenesis existed at the horizontal position. BSF-2 and above are considered to indicate bony fusion.

### Statistical analysis

SPSS 22.0 for Windows software was used for statistical analysis. The measurement data in accordance with a normal distribution are shown as the mean ± standard deviation. The VAS score, ODI index, JOA score and radiological measurement indexes of the two groups were compared with the analysis of variance of repeated measurement. If the spherical test was not satisfied, the Greenhouse‒Geisser method was used for correction. The general data, perioperative index, JOA score improvement rate, multifidus muscle atrophy rate and fusion rate between the two groups were compared with independent sample t tests. The α value of the test level is 0.05 on both sides. The counting data were expressed as rates, and the rank sum test or Fisher’s exact probability method was used for comparisons between groups. *P* < 0.05 was considered significant.

## Results

### Participants

A total of 22 patients from August 2017 to August 2019 were included in this study, including 14 males and 8 females. Patients aged 41–72 years (58.3 ± 2.9 years) were divided into two groups. OLIF with LSF and PETD was the OLIF + PETD group, including L_3_-L_4_ in 2 cases and L_4_-L_5_ in 8 cases. The control group received MIS-TLIF with percutaneous pedicle screw fixation (PPSF), including 12 patients, including L_3_-L_4_ in 1 case and L_4_-L_5_ in 11 cases. The patients were followed up for 12–22 months, with an average of 16 months. The patient data are summarized in Table 1.

**Table 1:** General information and perioperative indicators

	OLIF + PETD	MIS-TLIF	t	P
Number of patients	10	12		
Age (year)	60.5 ± 10.8	55.1 ± 11.3	1.143	0.266
Sex, n (%)				
Men	6(60%)	8(66.7%)		
Women	4(40%)	4(33.3%)		
Fusion segment, n (%)				
L_3_-L_4_	2(20%)	1(8.3%)		
L_4_-L_5_	8(80%)	11(91.7%)		
Operation time (min)	150.0 ± 22.0	171.7 ± 35.9	1.804	0.112
Incision length (mm)	6.3 ± 0.4	7.8 ± 0.4	0.056	< 0.001*
Hospital stays (d)	10.9 ± 5.0	10.8 ± 3.9	0.169	0.972
Intraoperative blood loss (ml)	117 ± 20.6	131 ± 60.4	2.452	0.474
Postoperative drainage (ml)	123.0 ± 41.0	127.1 ± 72.9	1.399	0.877

Values are the mean ± standard deviation

*OLIF* Oblique lumbar interbody fusion, *PETD* Percutaneous endoscopic transforaminal discectomy, *MIS-TLIF* Minimally invasive transforaminal lumbar interbody fusion

^*^p < 0.05, statistical significance

### General data and perioperative indicators

There was no significant difference in age, sex, operation segment, operation duration, hospital stays, intraoperative blood loss or postoperative drainage between the two groups. The total incision length in the OLIF + PETD group was significantly shorter than that in the MIS-TLIF group (*P* < 0.001, Table 1).

### Clinical efficacy evaluation

There were significant differences in the VAS score and ODI index at each time point in the two groups (*P* = 0.000). There was an interaction between the VAS score time and the group (F = 5.330, *P* = 0.009). With the extension of time, the decrease was greater in the OLIF + PETD group, and the VAS score in the OLIF + PETD group was lower than that in the MIS-TLIF group at 7 days after operation and at the last follow-up (*P* < 0.05). There was no significant difference between groups (F = 3.359, *P* = 0.082, Table 2). There was no interaction between ODI index time and group (F = 1.185, *P* = 0.327), and there was no significant difference between groups (F = 0.674, *P* = 0.421, Table 2).

**Table 2:** Clinical outcomes

	OLIF + PETD	MIS-TLIF	Statistic
Pre VAS	7.2 ± 0.8	7.1 ± 0.7	time F = 737.969, *P* = 0.000 time*group F = 5.330, *P* = 0.009 group F = 3.359, *P* = 0.082
Post VAS^#^	2.4 ± 0.5	3.1 ± 0.7	
F/U VAS	0.9 ± 0.6	1.6 ± 0.7	
Pre ODI	77.5 ± 11.5	74.7 ± 5.7	time F = 549.758, *P* = 0.000 time*group F = 1.185, *P* = 0.327 group F = 0.674, *P* = 0.421
Post ODI	16.4 ± 6.4	20.9 ± 4.9	
F/U ODI	9.8 ± 2.3	12.1 ± 4.0	
Pre JOA	9.2 ± 1.9	8.7 ± 1.9	time F = 630.159, *P* = 0.000 time*group F = 0.410, *P* = 0.666 group F = 0.330, *P* = 0.572
Post JOA	18.2 ± 2.0	18.4 ± 1.7	
F/U JOA	25.2 ± 0.9	24.8 ± 0.9	
Pre—Post improvement (%)	45.4 ± 9.7	47.8 ± 8.2	t =—0.654, *P* = 0.520
Pre—F/U improvement (%)	80.5 ± 5.8	79.0 ± 4.4	t = 0.711, *P* = 0.485

Values are the mean ± standard deviation

*VAS* Visual analog scale, *ODI* Oswestry Disability Index, *JOA* Japanese Orthopedic Association, *Pre* Pre-operation, *Post* 7 days after operation, *F/U* Final follow-up

^#^*P* < 0.05 OLIF + PETD group vs MIS-TLIF group

There were significant differences in JOA scores at each time point in the OLIF + PETD group and MIS-TLIF group (*P* < 0.001). There was no interaction between time and group (*P* > 0.05). There was no significant difference in JOA score between the two groups (*P* > 0.05, Table 2). There was no significant difference in the improvement rate of JOA score 7 days after operation and the last follow-up between the two groups (*P* > 0.05, Table 2).

### Degree of lumbar multifidus muscle injury

At the last follow-up, the rate of lumbar multifidus muscle atrophy in the OLIF + PETD group was 4.1 ± 0.5% and that in the MIS-TLIF group was 20.8 ± 3.6%, and the difference was statistically significant (*P* < 0.001, Table 3).

**Table 3:** Multifidus atrophy rate at the last follow-up

	MF-CSA		Atrophy rate (%)
	Pre	F/U^#^	Pre—F/U*
OLIF + PETD	7.7 ± 1.1	7.4 ± 1.1	4.1 ± 0.5
MIS-TLIF	7.9 ± 1.2	6.2 ± 0.8	20.8 ± 3.6
*P*	0.740	0.007	0.000

Values are the mean ± standard deviation

*MF-CSA* Multifidus muscle cross-sectional area, *Pre* Pre-operation, *F/U* Final follow-up

^*^P < 0.001 OLIF + PETD group vs MIS-TLIF group

^#^*P* < 0.05 OLIF + PETD group

### Radiological evaluation

**Fig. 7:**
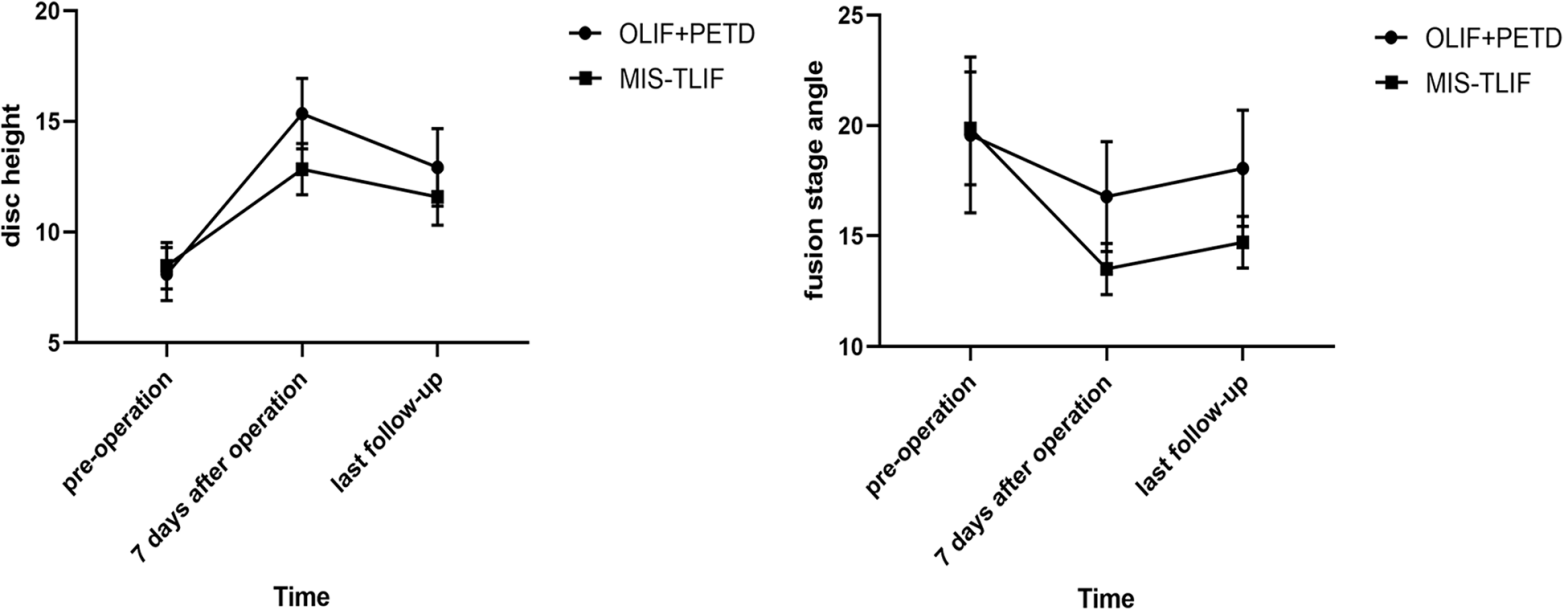
The trend of DH and FSA in the OLIF + PETD group and MIS-TLIF group

The lateral X-ray films of lumbar showed that there were significant differences in the DH at each time point in the OLIF + PETD group or MIS-TLIF group (F = 280.363, *P* = 0.000); there was an interaction between time and group (F = 16.836, *P* = 0.000), and the increase in DH was greater in the OLIF + PETD group with the passage of time (Fig. 7). There were statistically significant differences between the two groups. The DH in the OLIF + PETD group was higher than that in the MIS-TLIF group 7 days after the operation, but there was no significant difference before the operation and at the last follow-up (*P* > 0.05, Table 4). The difference in LA at each time point in the OLIF + PETD group or MIS-TLIF group was statistically significant (F = 128.416, *P* = 0.000); there was an interaction between time and group (F = 1.930, *P* = 0.173); and there were no statistically significant differences between the two groups (F = 0.342, *P* = 0.565, Table 4). The difference in FSA at each time point in the OLIF + PETD group or MIS-TLIF group was statistically significant (F = 72.579, *P* = 0.000); there was an interaction between time and group (F = 11.955, *P* = 0.000), and the decrease in FSA was greater in the MIS-TLIF group with the passage of time (Fig. 7). There were statistically significant differences between the two groups (F = 5.069, *P* = 0.036). The FSA in the OLIF + PETD group was higher than that in the MIS-TLIF group 7 days after the operation and at the last follow-up, but there was no significant difference before the operation (*P* > 0.05, Table 4).

According to lumbar CT measurements, there were statistically significant differences in bilateral FH and F-CSA at each time point in the MIS-TLIF group or OLIF + PETD group (*P* < 0.001); there was no interaction between time and group (*P* > 0.05); and there was no significant difference between the two groups (*P* > 0.05, Table 4).

Lumbar MRI measurements showed that there were statistically significant differences in SSCD, ASCD and SC-CSA at each time point in the MIS-TLIF group or OLIF + PETD group (*P* < 0.001); there was no interaction between time and group (*P* > 0.05); and there was no significant difference between the two groups (*P* > 0.05, Table 4).

### Degree of interbody fusion at last follow-up

**Fig. 8:**
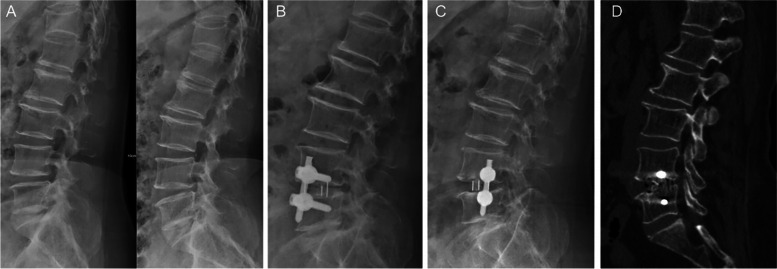
A 68-year-old male patient in the OLIF + PETD group was diagnosed with L_4-5_ lumbar disc herniation and lumbar instability. **A** Preoperative lumbar hyperextension and flexion radiographs showed lumbar instability. **B** Lumbar lateral X-ray 7 days after operation. **C** Lateral lumbar X-ray 16 months after surgery. **D** Lumbar CT at the last follow-up showed a completely continuous trabecular bone connecting the upper and lower endplates of the vertebral body in or outside the fusion cage (BSF-3)

At the last follow-up, 1 case in the OLIF + PETD group was BSF-1 grade (without fusion), and the fusion rate was 90%. In the MIS-TLIF group, 1 case was BSF-1 grade (without fusion), and the fusion rate was 91.7%. There was no significant difference in the fusion rate between the two groups (*P* > 0.05, Table 5). The fusion degree of a patient with OLIF + PETD at the last follow-up was shown in Fig. 8.

**Table 4:** Radiological outcomes

	OLIF + PETD	MIS-TLIF	Statistic
Pre DH	8.7 ± 1.2	8.5 ± 1.1	time F = 280.363, *P* = 0.000 time*group F = 16.836, *P* = 0.000 group F = 5.547, *P* = 0.030
Post DH*	15.3 ± 1.6	12.8 ± 1.2	
F/U DH	12.9 ± 1.7	11.6 ± 1.3	
Pre LA	36.2 ± 7.7	35.6 ± 3.2	time F = 128.416, *P* = 0.000 time*group F = 1.930, *P* = 0.173 group F = 0.342, *P* = 0.565
Post LA	50.5 ± 5.9	48.1 ± 4.2	
F/U LA	44.1 ± 4.8	43.7 ± 3.3	
Pre FSA	19.6 ± 3.5	19.9 ± 2.6	time F = 72.579, *P* = 0.000 time*group F = 11.955, *P* = 0.000 group F = 5.069, *P* = 0.036
Post FSA^#^	16.8 ± 2.5	13.8 ± 1.2	
F/U FSA^#^	17.6 ± 2.6	15.0 ± 1.2	
Pre RFH	9.9 ± 1.1	9.8 ± 1.9	time F = 195.346, *P* = 0.000 time*group F = 2.111, *P* = 0.134 group F = 0.737, *P* = 0.401
Post RFH	14.5 ± 1.8	15.5 ± 1.2	
F/U RFH	13.7 ± 1.6	14.1 ± 0.6	
Pre LFH	10.3 ± 1.3	9.8 ± 1.9	time F = 74.970, *P* = 0.000 time*group F = 0.125, *P* = 0.883 group F = 0.262, *P* = 0.614
Post LFH	14.8 ± 1.9	14.6 ± 0.8	
F/U LFH	13.7 ± 1.4	13.6 ± 1.0	
Pre RF-CSA	73.2 ± 12.1	76.6 ± 12.5	time F = 146.079, *P* = 0.000 time*group F = 0.454, *P* = 0.642 group F = 2.365, *P* = 0.140
Post RF-CSA	129.7 ± 7.4	136.5 ± 12.8	
F/U RF-CSA	123.7 ± 6.7	127.6 ± 7.9	
Pre LF-CSA	79.5 ± 12.9 143.3 ± 18.2 131.0 ± 18.6	80.7 ± 16.6	time F = 74.409, *P* = 0.000 time*group F = 1.204, *P* = 0.322 group F = 0.601, *P* = 0.447
Post LF-CSA		134.2 ± 16.1	
F/U LF-CSA		126.7 ± 13.8	
Pre SSCD	8.6 ± 1.9	8.5 ± 1.6	time F = 83.034, *P* = 0.000 time*group F = 0.104, *P* = 0.902 group F = 0.526, *P* = 0.477
Post SSCD	13.9 ± 1.0	13.6 ± 1.0	
F/U SSCD	13.1 ± 1.1	12.9 ± 0.8	
Pre ASCD	4.5 ± 1.6	5.5 ± 1.5	time F = 161.466, *P* = 0.000 time*group F = 0.257, *P* = 0.766 group F = 3.260, *P* = 0.086
Post ASCD	12.2 ± 1.7	12.7 ± 0.7	
F/U ASCD	11.3 ± 1.1	12.0 ± 1.2	
Pre SC-CSA	80.5 ± 19.5	76.9 ± 12.9	time F = 101.684, *P* = 0.000 time*group F = 0.532, *P* = 0.596 group F = 1.312, *P* = 0.266
Post SC-CSA	150.6 ± 18.4	145.3 ± 14.1	
F/U SC-CSA	143.5 ± 21.1	134.4 ± 13.3	

Values are the mean ± standard deviation

*DH* Disc height, *LA* Lumbar lordosis angle, *FSA* Fusion stage angle, *RFH* Right foraminal height, *LFH* Left foraminal height, *RF-CSA* Right foraminal cross section area, *LF-CSA* Left foraminal cross section area, *SSCD* Sagittal spinal canal diameter, *ASCD* Axial spinal canal diameter, *SC-CSA* Spinal canal cross-sectional area, *Pre* Pre-operation, *Post* 7 days after operation, *F/U* Final follow-up

*P < 0.001 OLIF + PETD group vs MIS-TLIF group

^#^*P* < 0.05 OLIF + PETD group vs MIS-TLIF group

**Table 5:** Fusion rate at last follow-up

	BSF grading			Fusion rate(%)
	BSF-1	BSF-2	BSF-3	Total
OLIF + PETD	1	7	2	9/10(90%)
MIS-TLIF	1	8	3	11/12(91.7%)
*P*				0.714

BSF-2 and above are considered to indicate bony fusion

### Complications

One of the 22 patients had postoperative complications. The patient had low back pain combined with radiating pain in the left lower limb for two years, was diagnosed with LDH combined with lumbar instability (L_4-5_) and was treated by OLIF + PETD in our hospital. The lower back pain and radiating pain in the left lower extremity were relieved after the operation, but numbness in the lower extremities still existed, and left thigh weakness and discomfort in the front of the thigh appeared. It was considered to be caused by intraoperative stretching of the psoas major muscle. The patient was given symptomatic treatment, such as nutritional nerve, anti-inflammatory and analgesic drugs, and asked to take the initiative to perform lower limb functional exercise in bed. The above symptoms disappeared after one week. One year after the operation, the symptoms of pain and numbness were significantly relieved in the outpatient follow-up.

## Discussion

In recent years, the methods of spinal fusion have increased, and minimally invasive technology (MIS) is booming in the field of spinal surgery to reduce tissue damage and pain. For example, XLIF allows restoring the sagittal and the coronal alignments, enlarging the nerve roots foramina, and providing higher stability that leads to that leads to posterior fusion of the lumbar facets [10]. MIS-TLIF was first proposed by Foley [11], which reduces the traction and injury of paraspinal muscles caused by conventional posterior lumbar surgery and reduces the incidence of complications related to the open surgical approach. Recently, however, Zhao [4] et al. reported that there was no significant difference in the VAS score and ODI index one month after MIS-TLIF compared with open TLIF, which was also proven by Li [3] et al. This may be because MIS-TLIF does not substantially avoid injury to the lumbar multifidus muscle. During the operation, the compression of the channel and long-term traction caused damage to the capillaries in the muscle, caused obvious ischemic changes, and aggravated the injury of the multifidus muscle [12]. The OLIF avoids the dissection of back muscles and damage to the bone structure by accessing the lumbar disc through the space between the aorta and psoas muscle and ensures the stability of the posterior column structure. This study shows that the rate of multifidus muscle atrophy at the last follow-up in the MIS-TLIF group was 20.8 ± 3.6% and that in the OLIF + PETD group was 4.1 ± 0.5%, so OLIF + PETD can avoid injury to the multifidus muscle during surgery. He [13] et al. also demonstrated that PPSF caused atrophy of the multifidus and erector spinae. Meanwhile, the VAS score of low back pain in the OLIF + PETD group was 2.4 ± 0.5 at 7 days after the operation and 0.9 ± 0.6 at the last follow-up, which were significantly lower than those in the MIS-TLIF group.

The OLIF is suitable for patients who need to re-establish intervertebral stability and restore intervertebral space height, among which lumbar instability is a good indication. The larger fusion cage used by OLIF can obtain a larger contact area with the endplate and better stability than the fusion cage used by MIS-TLIF. By effectively restoring the intervertebral space height, the posterior ligament and soft tissue are tightened and the displacement of the vertebral body is reduced. At the same time, indirect decompression is achieved by expanding the intervertebral space height and the intervertebral foramen area. However, due to the limited decompression effect, its application is limited. The PETD can remove the protruded or free lumbar intervertebral disc tissue and enlarge the intervertebral foramen under direct endoscopic vision by inserting the working channel directly into the spinal canal through the intervertebral foramen approach. The intervertebral foramen approach also has many advantages, such as protection of the posterior ligament and bone structure, less postoperative instability, facet joint disease and narrowing of the intervertebral space [14]. In addition, there is no chronic neuro edema or fibrosis that may be caused by interference with the epidural venous system. Epidural scar formation occurs in more than 10% of patients after open discectomy; however, this common sequela, which can lead to clinical symptoms, was not observed in PETD [15]. Therefore, PETD combined with OLIF can effectively ensure the effect of decompression and has significant advantages in small incision, low risk of postoperative trauma, short hospital stay and less blood loss [6, 16]. Recently, Yang [17] et al. retrospectively analyzed 19 patients with adjacent segment degeneration who were treated with PETD + OLIF or PLIF. The results showed that the PETD + OLIF group had a shorter operation time, less intraoperative bleeding and the lower postoperative VAS score.

Stand-Alone OLIF only uses a fusion cage without internal fixation. Biomechanical experiments show that subsidence of the lateral fusion cage easily occurs in the later stage of follow-up [18]. Tempel [19] et al. retrospectively analyzed 297 patients who underwent stand-alone OLIF and found that the application of a lateral fusion cage combined with internal fixation could further increase the stability of fusion. PPSF is routinely used in MIS-TLIF and OLIF techniques, and they are considered to be the standard internal fixation methods for the strongest spinal fixation [20, 21]. However, this kind of internal fixation will increase the total operation time and expenditure of patients [22]. Although it has been reported that placing pedicle screws in a lateral recumbent position can reduce the operation time, the risks outweigh the benefits [23]. The injury of paraspinal muscles caused by posterior fixation greatly also reduces the attractiveness of pedicle screw fixation. Liu [24] reported that OLIF combined with lateral vertebral screw fixation greatly reduced the operation time, intraoperative blood loss, radiation exposure and soft tissue injury and achieved one-stage interbody fusion through a single incision.

Recently, Xu [25] reported LSF after microsurgical nerve decompression via the OLIF approach, and the clinical effect was good. Wang [26] et al. reported that OLIF combined with LSF can correct both coronal and sagittal deformities in patients with moderate degenerative spinal deformities. Huang [27] et al. reported that compared with MIS-TLIF, OLIF significantly improved the VAS and ODI of patients with single-segment lumbar degenerative diseases and had significant advantages in the recovery of segmental lordosis and coronal imbalance. The results of this study showed that the clinical efficacy of the two groups was significantly improved 7 days after the operation, and the last follow-up VAS score, ODI index, JOA score and improvement rate were significantly improved. In addition, during the postoperative follow-up, the VAS score of the OLIF + PETD group was lower than that in the MIS-TLIF group, and injury to the multifidus muscle was avoided, indicating that OLIF + PETD has practical clinical value in clinical efficacy. X-ray results showed that although there was no significant difference in DH between the two groups at the last follow-up, OLIF's unique interbody fusion cage had certain advantages in the improvement of DH immediately after operation. Meanwhile, the CT and MRI results showed that the bilateral FH, F-CSA, SSCD, ASCD and SC-CSA were significantly improved 7 days after the operation and at the last follow-up, which also showed that the decompression effect of OLIF + PETD was similar to that of MIS-TLIF. With regard to the effect of fixation and fusion, the finite element analysis of Guo [21] based on a three-dimensional scanning model showed that the ability of lateral screw-rod internal fixation to limit the isotropic range of motion of lumbar vertebra was less than that of pedicle screws but better than stand-alone OLIF, especially in the direction of flexion and extension, while the improvement of cage stress by the lateral screw-rod technique was not as good as that of the pedicle screw technique but was significantly better than that of stand-alone OLIF. The results of this study showed that compared with the PPSF used by MIS-TLIF, the LSF used by OLIF + PETD had a less significant effect on the improvement of the FSA at 7 days after operation and at the last follow-up. This may be due to the lack of sagittal stability and the ability to restore the normal sequence provided by LSF, but the fusion rate reached 90% at the last follow-up, which proves that it can guarantee the fusion rate. Furthermore, in patients with lumbar deformities, the effect of these surgical correction on spinopelvic or lumbo-pelvic-femoral parameters should also be considered, such as Pelvic Index (PI), Pelvic Tilt (PT), Sacral Slope (SS), Sagittal Vertical Axis (SVA), Femoral Obliquity Angle (FOA) and T1 Pelvic Angle (TPA) [28], which could be important prognostic parameters for predicting disability and quality of life after spinal surgery.

In clinical application, it should be noted that although LSF takes into account both preserving the range of motion and improving the stress of the fusion cage to reduce the possibility of subsidence, in terms of the stability of the operative segment, LSF may be suitable for patients with normal bone mineral density and body mass index. In addition, in the process of screw placement, the tail of screws should be avoided to protrude the bone surface of the vertebral body, which may cause complications caused by peripheral vasculature, nerve injury and chronic stimulation of the psoas major muscle. Minimally invasive techniques are commonly used in lumbar tumor surgery [29]. Compared with open posterior instrumented fusion (OPIF), PPSF has fewer complications, lower infection rate, less intraoperative blood loss and shorter hospital stay [30]. Therefore, MIS-TLIF may be used for neuro-decompression and lumbar fusion to alleviate clinical symptoms in patients with Epidural Spinal Cord Compression (ESCC) grade 3 and underlying clinical or radiographic instability [29]. Telera [31] reported that OLIF can reduce the tissue trauma of patients, and achieve corpectomy and solid reconstruction of lumbar vertebral bodies. However, OLIF combined with LSF still needs further clinical studies. Moreover, compared with MIS-TLIF, OLIF + PETD can be used in patients with a history of posterior lumbar surgery, including open laminectomy and lumbar structural deformities, and the skin of the back is not suitable for posterior surgery. Under the premise of not increasing the operation time, a good surgical effect was achieved.

### Limitations

The limitations of this study are as follows: ① the number of cases is small, and a larger sample size is needed to study and demonstrate; ② the follow-up time is short, and the long-term effect still needs further clinical study. ③ all the cases come from a single medical center, which may cause some limitations; ④ without finite element analysis and evaluation, the stability of biomechanics still needs to be further studied.

## Conclusion

In summary, OLIF-PETD and MIS-TLIF can achieve satisfactory clinical results in the treatment of LDH with lumbar instability. However, compared with MIS-TLIF, OLIF-PETD has a smaller incision, less damage to the posterior bony structure, ligaments and muscles of the lumbar spine, relieves postoperative chronic low back pain, and has certain advantages in intervertebral space stretching. Therefore, OLIF-PETD may be an alternative choice for the treatment of LDH with lumbar instability.

## Data Availability

The datasets and materials supporting the conclusions of this article are included within the article, further inquiries can be directed to the corresponding author.
